# Jatrorrhizine Hydrochloride Suppresses RANKL-Induced Osteoclastogenesis and Protects against Wear Particle-Induced Osteolysis

**DOI:** 10.3390/ijms19113698

**Published:** 2018-11-21

**Authors:** Hui Li, Jing Wang, Qiwen Sun, Gang Chen, Shengnan Sun, Xuemei Ma, Haiwen Qiu, Xuerong Liu, Liangyi Xu, Mei Liu

**Affiliations:** Jiangsu Key Laboratory for Molecular and Medical Biotechnology, College of Life Sciences, Nanjing Normal University, Nanjing 210023, China; 15050527874@163.com (H.L.); njskywj@163.com (J.W.); sunqiwen123@163.com (Q.S.); m18852005775@163.com (G.C.); alyssia_s@163.com (S.S.); njnumaxuemei@163.com (X.M.); 15895937858@163.com (H.Q.); 13852240843@163.com (X.L.); 15651792179@163.com (L.X.)

**Keywords:** jatrorrhizine hydrochloride, osteoclast formation, bone resorption, wear particle-induced osteolysis, MAPK signaling pathway

## Abstract

Wear particle-induced aseptic prosthetic loosening is a major complication associated with total joint arthroplasty (TJA). A growing body of evidence suggests that receptor activator of nuclear factor κ-B ligand (RANKL)-stimulated osteoclastogenesis and bone resorption are responsible for peri-implant loosening. Thus, agents which attenuate excessive osteoclast differentiation and function have been considered to offer therapeutic potential for prolonging the life of TJA implants. Jatrorrhizine hydrochloride (JH), a major protoberberine alkaloid isolated from the traditional Chinese herb *Coptis chinensis*, has been reported to have antimicrobial, antitumor, and antihypercholesterolemic and neuroprotective activities. However, its effects on osteoclast biology remain unknown. Here, we found that JH inhibited RANKL-induced osteoclast formation and bone resorption in vitro and exerted protection against titanium (Ti) particle-induced osteolysis in vivo. Biochemical analysis demonstrated that JH suppressed RANKL-induced activation of MAPKs (p38 and ERK) which down-regulated the production of NFATc1 and NFATc1-regulated osteoclastic marker genes, such as *TRAP*, *CTR* and *CTSK*. Collectively, our findings suggest that JH may be a promising anti-osteoclastogenesis agent for treating periprosthetic osteolysis or other osteoclast-related osteolytic diseases.

## 1. Introduction

Total joint arthroplasty (TJA) is an effective procedure for treating end-stage joint diseases such as rheumatoid arthritis and osteoarthritis [1]. However, periprosthetic osteolysis and subsequent aseptic loosening is the leading complication which reduces the lifespan of implants [2,3]. Several strategies have been developed to overcome this problem, including improving the implant designs, perfecting surgical techniques, and searching for better biomaterials. However, these strategies are unlikely to completely eliminate particle generation from implants [4,5]. Despite the precise mechanisms remaining unclear, wear particle-triggered over-activated osteoclastogenesis and extensive bone erosion have been demonstrated to be vital etiologies in periprosthetic osteolysis [6,7]. During the last two decades, many agents have been demonstrated to target osteoclast function and suppress wear debris-stimulated bone resorption, such as bisphosphonates [8,9], erythromycin [10] and denosumab [11]. However, their long-term use could cause severe side effects, including digestive system disorders, osteonecrosis, and even some types of malignancy [12,13,14,15]. Therefore, it is still of great importance to explore alternative drugs with less side effects to address these problems.

Jatrorrhizine hydrochloride (JH) is one of the major protoberberine alkaloids isolated from the herb *C. chinensis*, and has been reported to possess some pharmacological properties, such as bacteriostasis [16,17], antioxidation [18], tumor cell growth suppression [19,20], and antidiabetic and antihyperlipidemic activities [21,22]. Nevertheless, to our knowledge, no study has investigated its anti-osteoclastogenic and anti-resorptive actions. In our recently published paper, JH has been demonstrated to inhibit focal bone erosion in collagen-induced arthritis (CIA) rat model [23]. As osteoclast is the sole cell type responsible for bone resorption, therefore, we speculated that JH may have a potential therapeutic role of anti-osteolytic diseases through inhibiting osteoclastogenesis. We tested this possibility in the current study, using a Ti particle-induced murine calvarial osteolytic model in vivo and RANKL-induced osteoclastogenesis in vitro, and subsequently investigated their underlying mechanisms.

## 2. Results

### 2.1. Therapeutic Effect of JH on Ti Particle-Induced Osteolysis In Vivo

**Figure 1:**
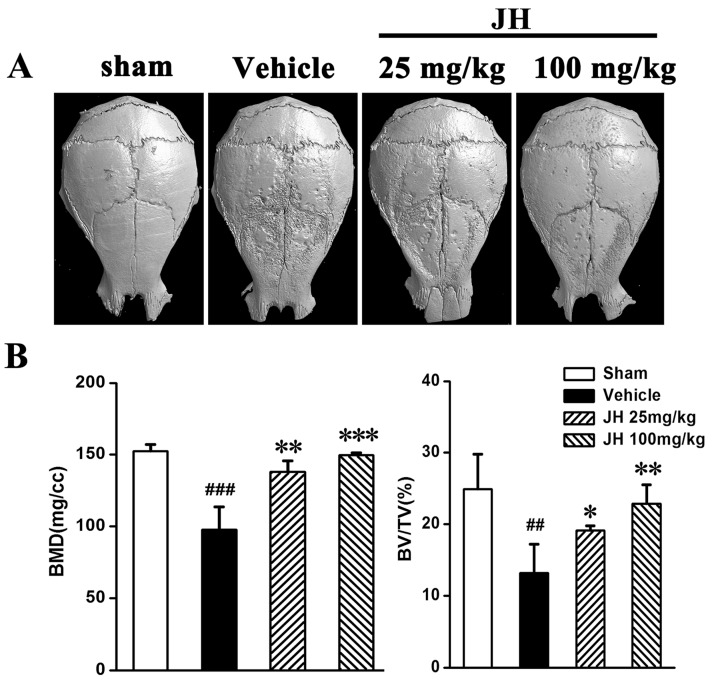
JH inhibits Ti particle-induced bone destruction in a mouse calvarial model. (**A**) Representative micro-CT 3D reconstructed images were obtained from different groups. (**B**) Bone mineral density (BMD) and bone volume/tissue volume (BV/TV) were determined using the CTan program (SkyScan). All the data were shown as mean ± SD; ## *p* < 0.01 and ### *p* < 0.001 versus sham group, * *p* < 0.05, ** *p* < 0.01 and *** *p* < 0.001 versus vehicle-treated Ti particle-induced group; *n* = 7 per group.

To investigate the possible inhibitory effect of JH on pathological osteolysis in vivo, a Ti particle-induced murine calvarial osteolytic model was established. Three-dimensional reconstruction data revealed that, compared to the sham group, Ti particles induced severe osteolysis, as evidenced by extensive erosion on the bone surface of the vehicle group (Figure 1A). In contrast, JH significantly and dose-dependently reduced the severity of Ti particle-induced osteolysis. Bone loss in the high-dose JH treated group was markedly less than that in the low-dose group (Figure 1A). Also, our quantitative µCT analysis showed that, compared to the vehicle group, JH treatment significantly increased bone mineral density (BMD) as well as bone volume/tissue volume (BV/TV) (Figure 1B), which further confirmed the reconstruction data.

**Figure 2:**
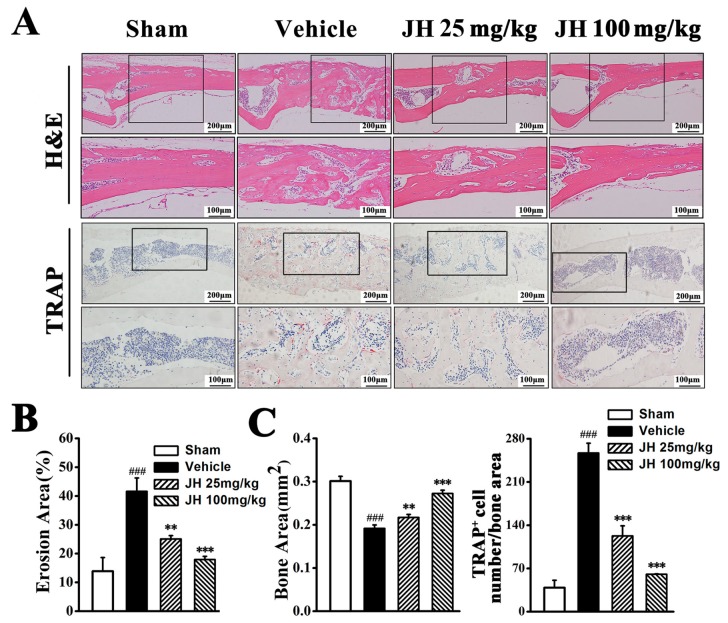
Histological staining and histomorphometric assessment of the inhibitory actions of JH on Ti particle-induced osteolysis. (**A**) Representative micrographs staining for H&E and tartrate-resistant acid phosphatase (TRAP). The regions marked by the black frames were enlarged and shown in the corresponding bottom panels. (**B**) The percentage of total porosity obtained from the H&E-stained sections. (**C**) The bone area (mm^2^) and TRAP^+^ osteoclast number in TRAP-stained sections were calculated and analyzed as described in the Section 4. All data were shown as mean ± SD; ### *p* < 0.001 versus sham group, ** *p* < 0.01 and *** *p* < 0.001 versus vehicle-treated Ti particle-induced group.

Histological assessment and histomorphometric analysis further demonstrated the amelioration of Ti particle-induced osteolysis by JH. Hematoxylin and eosin (H&E) staining showed that bone erosion had clearly occurred in the vehicle group, whereas JH treatment significantly reduced these osteolytic changes (Figure 2A). This result was confirmed by histomorphometric analysis. As shown in Figure 2B, both low and high doses of JH significantly reduced the erosive surface. Furthermore, tartrate-resistant acid phosphatase (TRAP) staining showed that, in the vehicle-treated group, the number of TRAP^+^ osteoclasts mainly distributing along the eroded surface was significantly increased. This increase was effectively decreased by JH treatment (Figure 2C), indicating that the inhibitory effect of JH on Ti particle-induced bone erosion was associated with the formation and function of osteoclasts.

In addition to efficiency, safety issues need to be addressed. In this study, JH-treated groups displayed normal behavioral activities and no obvious adverse effects or mortality occurred. Also, we examined the body weight and serum levels of alanine aminotransferase (ALT) and aspartate aminotransferase (AST) in JH treatment groups. As shown in Table 1, there was almost no body change in the JH-treated mice, as compared with the untreated Ti particle-induced mice and the control mice. In addition, JH-treated mice did not show any significant changes in serum ALT and AST levels, as compared with the untreated mice, further demonstrating that the present JH treatment had no significant adverse side effects.

### 2.2. JH Inhibited RANKL-Induced Osteoclast Formation In Vitro

**Figure 3:**
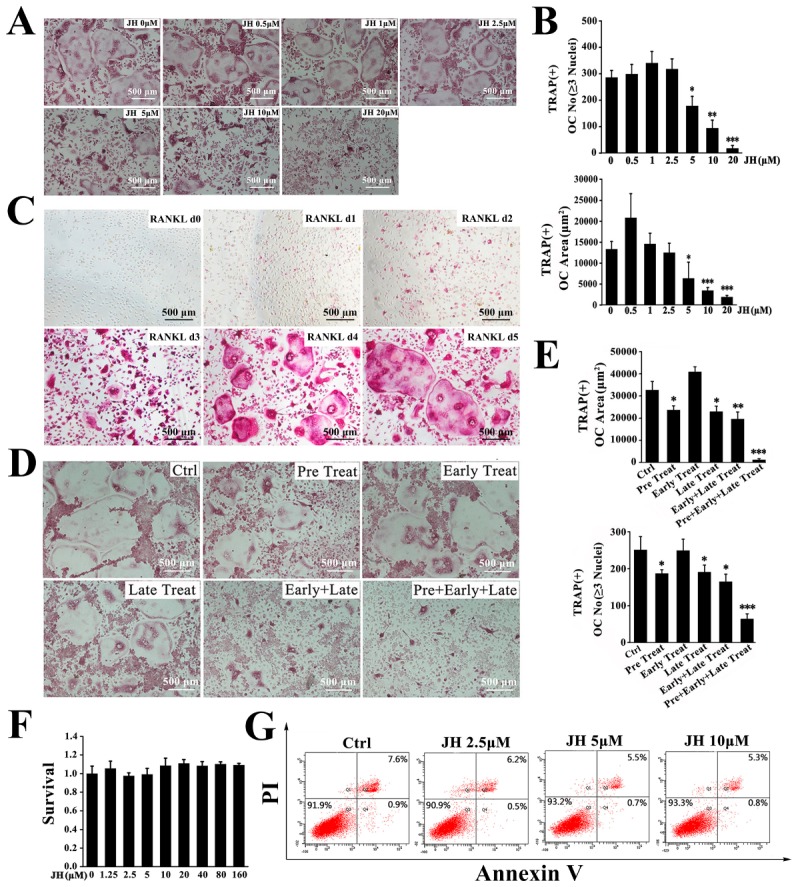
JH inhibits osteoclast formation in vitro. (**A**) A representative image showing the inhibitory effect of JH on osteoclast formation. BMM cells were treated with different concentrations of JH in the presence of RANKL (100 ng·mL^−1^) for 5 days and then TRAP staining was performed. The number and area of TRAP^+^ multinucleated cells with more than three nuclei were shown in (**B**), *n* = 3, * *p* < 0.05, ** *p* <0.01 and *** *p* < 0.001 relative to RANKL-treated, JH-untreated controls. OC No: osteoclast number. (**C**) To define the different stages of osteoclastogenesis, TRAP staining was performed on d0–d5 of RANKL induction (d0, the day RNAKL was added; d1, RANKL induction for 1 day; d2, RANKL induction for 2 days; and so on). (**D**) BMMs were cultured in the presence of RANKL, and JH (5 µM) was added in different times. At the end of 5 days, TRAP staining was performed. (Ctrl group: RANKL induction for 5 days without JH; Pre Treat group: JH pre-treatment for 12 h and RANKL induction for 5 days; Early Treat group: Co-treatment with JH and RANKL for 2 days and RANKL continued induction for 3 days; Late Treat group: RANKL induction for 2 days and subsequent co-treatment with JH and RANKL for 3 days; Early + Late Treat group: Co-treatment with JH and RANKL for 5 days; Pre + Early + Late Treat group: JH treatment for 12 h and subsequent co-treatment with JH and RANKL for 5 days) (**E**) The area and number of TRAP^+^ osteoclasts (≥3 nuclei) from (**D**) were quantified (*n* = 3). All data were shown as mean ± SD; * *p* < 0.05, ** *p* <0.01 and *** *p* < 0.001 relative to RANKL-treated, JH-untreated controls. (**F**) Effect of JH on cell viability was measured using a MTS assay. (**G**) BMMs were exposed to the indicated concentrations of JH for 24 h, and then cell apoptosis was determined using flow cytometry.

To investigate the mechanisms through which JH inhibited Ti particle induced-osteolysis, the effect of JH on osteoclast formation was determined in vitro. Bone marrow-derived macrophages (BMMs) were treated with different concentrations of JH for 5 days in presence of RANKL. In the control group, numerous TRAP-positive multinucleated osteoclasts were formed. However, JH at the dosages 5–20 µM significantly and dose-dependently inhibited the number and area of osteoclasts (Figure 3A,B). To investigate at which stage JH inhibited osteoclastogenesis, we firstly performed TRAP staining on d0–d5 of RANKL induction to define the different stages of osteoclastogenesis. As shown in Figure 3C, TRAP-positive mononuclear osteoclasts were mainly formed at the first two days of RANKL stimulation. At day 3 of RANKL induction, the multinucleated cells (MNCs) were obviously observed, and the number and size of TRAP^+^ MNCs were gradually increased with RANKL continuous treatment for 4–5 days. Thus, JH was added to osteoclast differentiation cultures at day 0 (the day RANKL was added), day 2 (RANKL induction for 2 days) and 12 h prior to RANKL stimulation. As shown in Figure 3D and 3E, JH treatment on the first two days of RANKL induction (Early Treatment) did not affect osteoclast formation, however, JH treatment 12 h prior to RANKL induction (Pre-Treatment) modestly but statistically significantly inhibited osteoclastogenesis. This is an interesting observation, and the involved molecular mechanisms remain to be clarified. In addition, JH treatment on day 3 of the 5 day culture (Late Treatment) modestly reduced the number or area of osteoclasts formed. Consecutive treatment with JH, for example 2 stages (Early and late) or 3 stages (Pre, Early and Late), showed a strong inhibitory effect on osteoclast formation (Figure 3D,E). To exclude the potential cytotoxic effect of JH on BMMs, a cell survival assay was performed. Our results showed that BMM viability or proliferation was not affected by JH treatment, even at concentration as high as 160 μM. (Figure 3F). To investigate the possible effect of apoptosis on osteoclast formation, an apoptosis assay was performed by flow cytometry. As shown in Figure 3G, concentrations of JH which were effective at inhibiting osteoclastogenesis did not affect apoptosis, indicating the inhibitory action of JH on osteoclastogenesis was not due to apoptosis.

### 2.3. JH Impaired Ostoclastic Bone Resorption and F-Actin Ring Formation In Vitro

**Figure 4:**
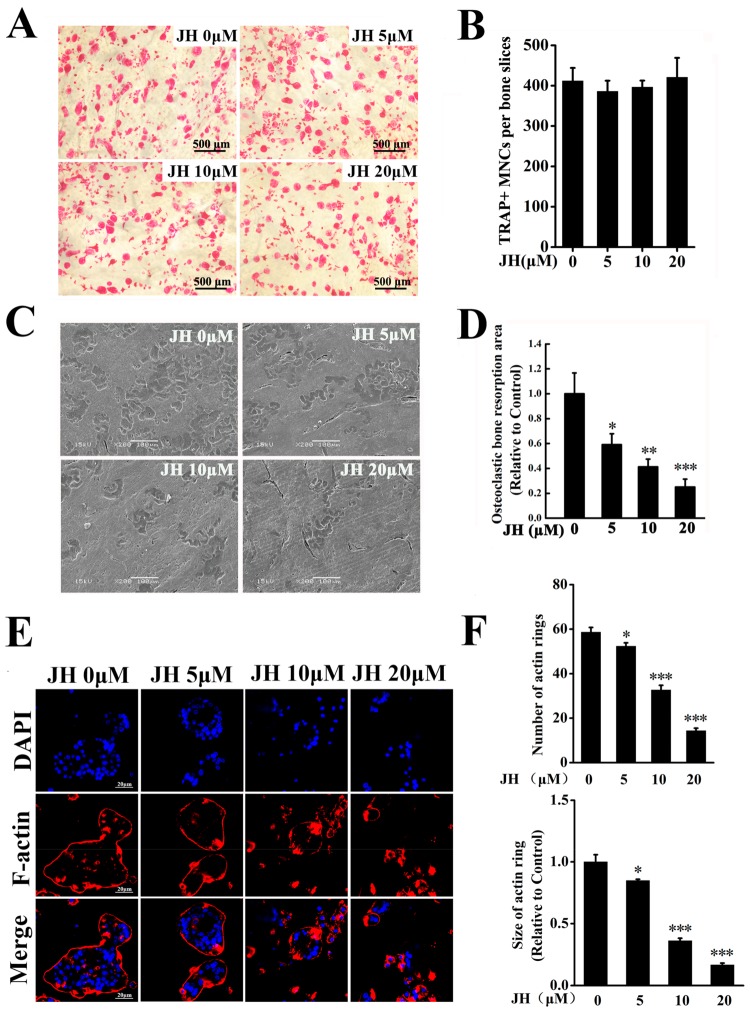
JH inhibits osteoclast-mediated bone resorption. (**A**) Representative TRAP staining images showing no cytotoxic effect of JH on mature osteoclasts. (**B**) The number of TRAP^+^ osteoclasts (≥ 3 nuclei) from (**A**) were quantified (*n* = 3). (**C**) Representative scanning electron microscopy (SEM) images showing the inhibitory effect of JH on osteoclast-mediated bone resorption. (**D**) Resorption area relative to the total area of bone was measured using ImageJ (*n* = 3). (**E**) Representative immunofluorescence staining images showing the inhibitory effect of JH on F-actin ring formation. (**F**) The number and size of intact actin rings from (**E**) were quantified (*n* = 3). * *p* < 0.05, ** *p* < 0.01 and *** *p* < 0.001 relative to RANKL-treated, JH-untreated controls.

Given that JH can inhibit osteoclast differentiation, we used mature osteoclasts to investigate the effect of JH on resorptive function. As shown in Figure 4A,B, treating mature osteoclasts with vary concentrations of JH did not alter the viability of cells, indicating that JH had no cytotoxic effect on mature osteoclasts. The resorptive pits on the bone slices were scanned by scanning electron microscopy. As expected, mature osteoclasts extensively resorbed bone matrix in the control group, however, JH significantly and dose-dependently attenuated osteoclast-mediated bone resorption (Figure 4C,D).

A well-polarized F-actin ring is well known to be a prerequisite for osteoclastic resorption. To further confirm the effect of JH on bone resorption, we investigated whether JH can affect F-actin ring formation. A shown in Figure 4E,F, well-structured F-actin rings were found in the control group. However, JH significantly and dose-dependently blocked F-actin ring formation, as evidenced by the decreased number and size of F-actin rings in JH-treated groups. These data indicated that the inhibitory effect of JH on osteoclastic resorption could partially be the result of impairing F-actin ring formation.

### 2.4. JH Inhibited RANKL-Induced Osteoclast-Specific Gene Expression

**Figure 5:**
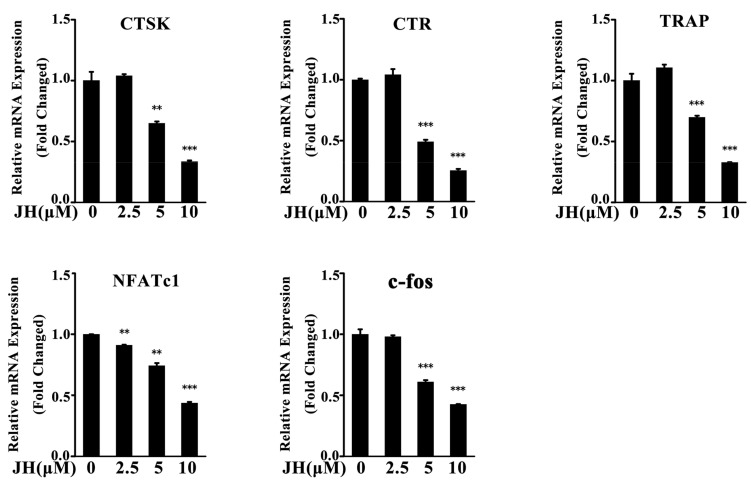
JH suppresses RANKL-induced osteoclast-specific gene expression during osteoclastogenesis. BMMs were treated with RANKL and varying concentrations of JH for 5 days. The levels of osteoclast-specific marker genes were analyzed by real-time PCR, normalized to *β-actin* expression (*n* = 3). All data were shown as mean ± SD; ** *p* < 0.01 and *** *p* < 0.001 relative to RANKL-treated, JH-untreated controls. CTSK: cathepsin K; CTR: calcitonin receptor; TRAP: tartrate-resistant acid phosphatase.

To further evaluate the inhibitory effect of JH on osteoclastogenesis, real-time PCR was performed. As shown in Figure 5, the expressions of osteoclast-specific genes including *cathepsin K* (*CTSK*), *calcitonin receptor* (*CTR*), *TRAP*, *nuclear factor of activated T-cells cytoplasmic 1* (*NFATc1*) and *c-fos* were significantly suppressed by JH at day 5 of culture. These data were consistent with the results of osteoclast formation and bone-resorptive activity assays.

### 2.5. JH Inhibited RANKL-Induced MAPK and NFATc1 Activation

**Figure 6:**
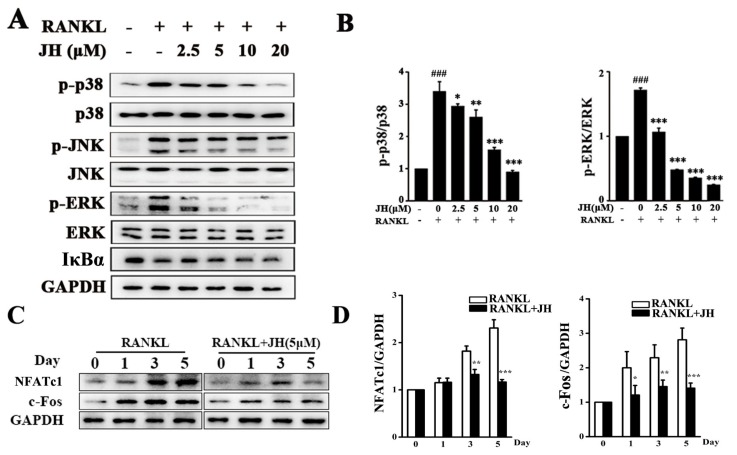
JH attenuates RANKL-stimulated activations of MAPKs (p38 and ERK). (**A**,**C**) After pretreatment with or without JH for 1 h, BMM cells were stimulated with RANKL (100 ng∙mL^−1^) for 15 min in (**A**), and 0, 1, 3, 5 days in (**C**). Western blotting was performed to examine the levels of p-p38, total p38, p-ERK1/2, total ERK1/2, p-JNK, total JNK, IκBα, NFATc1, c-Fos and GAPDH. (**B**,**D**) The ratios of p-p38/p38, p-ERK/ERK, NFATc1/GAPDH and c-Fos/GAPDH were quantified and calculated using ImageJ software (*n* = 3). All data were shown as mean ± SD; * *p* < 0.05, ** *p* < 0.01 and *** *p* < 0.001 relative to RANKL-treated, JH-untreated controls. ### *p* < 0.001 relative to RANKL-untreated, JH-untreated controls.

To explore the underlying mechanism through which JH-mediated osteoclast formation, RANKL-induced MAPK and NF-κB signaling pathways were investigated. As shown in Figure 6A,B, RANKL stimulation significantly increased the phosphorylation of the MAPK family members including extracellular signal-regulated kinase (ERK), c-Jun N-terminal kinase (JNK) and p38 kinase. The increases of p-p38 and p-ERK1/2 were significantly and dose-dependently inhibited by JH treatment. However, JH had no obvious effect on phosphorylation of JNK and degradation of IκBa, indicating that it does not influence the JNK and NF-κB pathways.

Activations of the MAPK (p38 and ERK) pathways subsequently lead to the induction and activation of NFATc1, which has been reported to be a master transcription factor for osteoclast differentiation and function [24]. Thus, we investigated whether JH affected the induction of NFATc1. As shown in Figure 6C,D, the protein level of NFATc1 was increased when cells were exposed to RANKL, and JH significantly attenuated this increase. Since c-fos has been reported to bind to the promoter of NFATc1 and thereafter regulate its expression [25], we next examined the protein level of c-fos. As expected, the level of c-fos was significantly decreased by JH treatment, further confirming the inhibitory action of JH on RANKL-induced NFATc1 expression (Figure 6C,D).

## 3. Discussion

To our best knowledge, the pharmacological studies of JH are very limited. Only few published papers have been found to report its bacteriostasis [16,17], tumor cell growth suppression [19,20], and antidiabetic and antihyperlipidemic activities [21,22]. Most of these reported pharmacological properties have been characterized based on animal models, and their involvements in cellular and molecular mechanisms are still not determined. In this study, we found a novel pharmacological function of JH, that is, it could effectively prevent wear particle-induced osteolysis in vivo and inhibit RANKL-induced osteoclastogenesis in vitro. And these inhibitory effects of JH might be mediated via the suppressed activation of MAPK (p38 and ERK).

In vivo studies showed JH significantly and dose-dependently suppressed Ti particle-induced bone destruction, as illustrated by significantly enhanced BMD and BV/TV. These results were further confirmed by histological analysis of tissues stained with H&E and TRAP. The vehicle group showed significant increases in erosion area and number of TRAP-positive multinucleated osteoclasts, whereas these increases were effectively decreased by JH treatment, indicating that JH could exert a therapeutic effect to treat Ti particle-induced osteolysis through inhibiting osteoclast formation and function. Indeed, our in vitro results clearly demonstrated that JH could directly suppress the number and area of TRAP positive cells stimulated by RANKL. In addition, a significant decrease in the bone resorption activity was also observed in JH-treated groups on the basis of pit formation on bone slices, determined by a scanning electron microscope.

Having established that JH plays inhibitory roles in Ti particle-induced osteolysis model and RANKL-induced osteoclastogenesis, we further investigated the underlying molecular mechanisms. It is well known that RANKL interacts with its receptor RANK to induce the recruitment and activa-tion of an important adaptor protein, TRAF6. Activated TRAF6 complexes with TAK1-RACK-MKK6 or TAK-TABs to induce the activation of MAPK including p38, ERK and JNK. Also, TRAF6 can stimulate NF-κB activity either by interaction with p62 and αPKC or via the regulation of TAK1-dependent phosphorylation [26]. Activated MAPK and NF-κB have been reported to play key roles in RANKL-mediated osteoclastogenesis and function [25,27,28,29,30,31]. In our study, JH did not affect JNK and NF-κB signaling pathways, however, it could significantly suppress the phosphorylation levels of RANKL-mediated p38 and ERK. Interestingly, our previous study showed that, in synoviocyte cell line MH7A, JH also significantly inhibited TNFα-induced p38 and ERK activation [23]. Thus, it is possible that blocking p38 and ERK activation might be an important active site of JH. Certainly, further investigations are needed to unveil its direct binding sites.

Following activation of RANKL-stimulated MAPK signaling cascades, a series of downstream transcription factors are induced [31,32,33]. Among them, NFATc1 is considered the most important transcription factor required for the regulation of osteoclastogenesis. In this study, JH was demonstrated to significantly inhibit the induction of NFATc1, which was further supported by the decreased expression of c-fos and down-regulation of NFATc1-regulated osteoclastic marker genes, such as *TRAP*, *CTR* and *CTSK* by JH.

Throughout this study, JH was well-tolerated at the given doses without obvious drug toxicity to liver function in the treated mice. Additionally, our in vitro study showed that up to 160 μM did not affect BMMs viability, suggesting that JH is a promising alternative medicine for osteoclast-related osteolytic therapy because of its efficiency and safety.

Despite these interesting findings, the present study has some limitations. Firstly, bone metabolism is a delicate balance of bone formation by osteoblasts and bone resorption by osteoclasts [34]. Bone formation has also been demonstrated to play an important role in osteolysis treatment [35,36]. It has been reported that, in addition to osteolysis, bone formation and repair of the osteolytic areas occur on the calvaria of Ti particle-induced osteolysis model [37]. In this study, the calvaria thickness in the vehicle group was observed to increase compared to that in the sham group, and this enlargement was gradually suppressed by JH treatment (Figure 2A). We speculated that the increased calvaria thickness was likely caused by bone formation and repair, and JH might play an inhibitory role in this process. Certainly, further investigations are needed to verify JH’s action on osteoblasts and bone formation. Secondly, the main cause of clinical periprosthetic osteolysis is polyethylene rather than Ti particles [38]. However, Ti particles are well-characterized. Furthermore, both Ti and polyethylene have been reported to comparably activate osteoclast differentiation and bone resorption to induce osteolysis in vivo [39,40]. Thus, both animal models are suitable to be used for investigating the inhibitory action of JH on osteolysis. Thirdly, the murine calvarial model is a pathological model but not a disease model. The lack of implant and mechanical load, and a limited two-week duration of the osteolytic process should be considered in this study. Despite these limitations, our study clearly demonstrated that JH exerted inhibitory actions on osteoclast formation and function in vitro and in vivo, indicating that this compound may be a therapeutic candidate for treating osteolysis.

## 4. Materials and Methods

### 4.1. Animals

A total of 28 healthy 8-week-old C57BL/6 mice were purchased from Beijing Vital River Laboratory Animal Technology Co. Ltd. (Beijing, China). They were housed under specific pathogen-free (SPF) conditions (22 °C, 12 h/12 h light/dark, 50–55% humidity) and given free access to food and water. All the animal experiments were approval by the Experimental Committee of Nanjing Normal University (#20160617, approved date: 7 June 2016).

### 4.2. Media and Reagents

Jatrorrhizine hydrochloride (C_20_H_20_ClNO_4_, Purity ≥ 98%) was purchased from Nanjing Zelang Medical Technology Co. Ltd. (Nanjing, Jiangsu, China). Alpha modified Minimal Essential Medium (α-MEM) and fetal bovine serum (FBS) were purchased from Gibco (Gibco BRL, Grand Island, NY, USA). Recombinant mouse RANKL and M-CSF were provided by PeproTech (Rocky Hill, NJ, USA). MTS reagents were obtained from Sigma-Aldrich (St. Louis, MO, USA). TRIzol reagent was obtained from Invitrogen (Carlsbad, CA, USA). Primary anti-bodies targeting phosphorylated (p) ERK, p-p38, p-JNK1/2, ERK, p38, JNK1/2, IκBα and GAPDH were obtained from CST (Cell Signaling Technology, Inc., Beverly, MA, USA). Anti-NFATc1 and anti-c-fos antibodies were purchased from BD Biosciences (San Jose, CA, USA). Commercial kits for ALT and AST measurement were from Jiancheng Institute of Biotechnology (Nanjing, Jiangsu, China). Tartrate-resistant acid phosphatase (TRAP) staining kits were obtained from Sigma-Aldrich.

Titanium (Ti) particles (1~3 μm diameter) were purchased from Johnson Mathey Chemicals (Ward Hill, MA, USA). The particles were baked for 6 h at 180 °C, and then treated with 70% ethanol for 48 h. The sterile particles were stored in sterile PBS (300 mg∙mL^−1^) at 4 °C until required for use.

### 4.3. Titanium Particle-Induced Calvarial Osteolysis Model

To measure the osteolysis-suppressing effect of JH in vivo, we established a Ti particle-induced calvarial osteolysis model, as described by the previous studies [41,42]. Briefly, 28 male eight-week-old C57BL/6 mice were randomly divided into four groups (*n* = 7): PBS control (sham), Ti particles in PBS (vehicle), Ti particles together with different doses of JH (25 mg∙kg^−1^∙day^−1^ and 100 mg∙kg^−1^∙day^−1^, respectively). The doses of JH were determined according to the previous studies [21,22] with modification from our preliminary experiments. The mice were anesthetized with avertin (300 mg∙kg^−1^ body weight), and the cranial periosteum was separated from the calvarium by sharp dissection. Thirty mg of Ti particles were then embedded under the periosteum at the middle suture of the calvaria. Two days after implantation, JH or PBS was intragastrically administrated every day for 14 days. At the end of the experiment, the mice were sacrificed and the serum was collected for ALT and AST analysis. And the calvaria were excised for micro-computerized tomography (CT) scanning and histological analysis.

### 4.4. Serum ALT and AST Measurement

Blood samples were harvested by heart puncture. After centrifugation, the supernatant was collected for ALT and AST measurement using the commercial kits according to the manufacturer’s protocols.

### 4.5. Micro-CT Scanning

The calvaria were removed and fixed in 4% paraformaldehyde for 48 h. After removal of the particles, the osteolysis in mouse calvariae was analyzed using a high-resolution micro-CT (SkyScan 1176, Bruker, Germany) with an isometric resolution of 9 µm. After reconstruction, we chosen a round region of interest (ROI) around the midline suture (4 mm diameter) for further quantitative analysis. BMD and BV/TV were measured using the CTan program (SkyScan, Kontich, Belgium).

### 4.6. Histological and Histomorphometric Analysis

After micro-CT scanning, the samples were decalcified, embedded, and stained with H&E and TRAP. The bone area and the percentage of total porosity were calculated with Image Pro-Plus 5.0 software (Media Cybernetics, Silver Spring, MD, USA). The number of TRAP^+^ osteoclasts were counted and normalized to bone area.

### 4.7. Cell Viability Assay

To examine the effect of JH on the viability of BMMs, a MTS assay was performed. BMMs were seeded in 96-well plates at a density of 6 × 10^3^ cells/well and cultured overnight to adhere. Varying concentrations of JH were added to the cells and incubated for 48 h. MTS/PMS mixture was then added and continuously cultured for another 4 h according to the manufacturer’s instructions. Absorbance was measured at 490 nm using a microplate reader (BMG LABTECH GMBH, Ortenberg, Germany). The effect of JH on cell viability was expressed as percent cell viability with vehicle treated control cells set at 100%.

### 4.8. In Vitro Osteoclastogenesis Assay

In vitro osteoclastogenesis assay was performed to examine the effect of JH on osteoclast differentiation. BMM cells were isolated, cultured and induced to differentiation as previously described [43]. Briefly, bone marrow cells isolated from femurs and tibias of 8-week-old C57BL/6 mice were incubated in complete cell culture media containing 30 ng·mL^−1^ M-CSF in a T-75 cm^2^ flask for proliferation. When confluent, BMMs were seeded into 96-well plates at a density of 6000 BMMs/well (in triplicate) and incubated overnight. BMMs were then treated with various concentrations of JH plus RANKL (100 ng·mL^−1^) and M-CSF (30 ng·mL^−1^). After five days, cells were fixed and stained for TRAP activity. TRAP^+^ multinucleated cells with more than three nuclei were counted as osteoclasts.

### 4.9. Bone Resorption Pit Assay and F-Actin Ring Formation Assay

Bone resorption assay was performed to test the effect of JH on osteoclast function. Bovine cortical bone slices (100 µm) were cut using a cutting machine (IsoMet Low Speed Saw; Buehler Ltd., Lake Bluff, IL, USA). After sonication and extensive washing with MilliQ water, the bone slices were sterilized with 70% ethanol for 2 min. The sterile bone slices were rinsed with sterile PBS for three times and then put into the 96-well plates to incubate for 4 h with α-MEM at 37 °C in 5% CO_2_ atmosphere. After removal of the medium, BMM were seeded on the bone slices at a density of 8000 cells per well (in triplicate) and incubated with M-CSF (30 ng·mL^−1^) and RANKL (100 ng·mL^−1^) for 3–4 days. When osteoclasts began to form, varying concentrations of JH were added and continuously cultured for another 2 days. The bone slices were then fixed with 4% paraformaldehyde and stained for TRAP activity. The number of TRAP positive multinucleated cells with more than three nuclei was counted. After gentle brushing the osteoclasts, the bone resorption pits were visualized by scanning electron microscopy (JSM 5610 LV; JEOL, Tokyo, Japan) and five view fields were randomly selected for each bone slice for further analysis. The areas of bone resorbed were determined with Image Pro-Plus 5.0 software (Media Cybernetics, Silver Spring, MD, USA). The result was shown as resorption area relative to the total area of bone. Experiments were repeated independently at least three times.

For F-actin ring formation assay, the experimental protocols for RANKL induction and JH treatment of BMM cells were consistent with those for bone resorption assay. Mature osteoclasts cultured on the bone slices were fixed with 4% paraformaldehyde for 15 min and then permeabilized with 0.5% Triton X-100 for 10 min. F-actin rings were incubated with rhodamine-conjugated phalloidin for 15 min and nuclei with DAPI dye. Fluorescence images were acquired using a Nikon A1R resonance scanning confocal microscope (Nikon, Tokyo, Japan). The number and size of F-actin rings were analyzed using ImageJ software (NIH, Bethesda, MD, USA).

### 4.10. Apoptosis Analysis by Flow Cytometry

Apoptosis assay was performed using an Annexin V-FITC apoptosis detection kit (KenGEN Biotech. Co. Ltd., Nanjing, Jiangsu, China) according to the manufacturer’s protocol. In brief, BMMs were exposed to JH (0, 2.5, 5, 10 µm) for 24 h and then suspended in binding buffer. The cells were stained with Annexin V and propidium iodide (PI), and the results were analyzed with FACScan (Becton Dickinson, Franklin Lakes, NJ, USA) with the CellQuest program.

### 4.11. RNA Isolation and Real-Time PCR Analysis

BMMs were seeded in 12-well plates at a density of 4 × 10^4^ cells/well. Cells were treated with different concentrations of JH (0, 2.5, 5, 10 µm) for 5 days in the presence of M-CSF (30 ng∙mL^−1^) and RANKL (100 ng∙mL^−1^). Total RNA was extracted and cDNA was synthesized from 500 ng of total RNA using reverse transcriptase (TaKaRa Biotechnology Co., Ltd., Dalian, Shenyang, China). Real-time PCR was conducted using Mastercycler ep realplex 2 systems (Eppendorf, Hamburg, Germany) with a SYBR Premix Ex Tag kit (TaKaRa Biotechnology). Three independent experiments were conducted. And each experiment was performed in triplicate. The following real-time PCR primers were used: *TRAP*, 5′-CTGGAGTGCACGATGCCAGCGACA-3′ (forward), 5′-TCCGTGCTCGGCGATGGACCAGA-3′ (reverse); *CTSK*, 5′-CTTCCAATACGTGCAGCAGA-3′ (forward), 5′-TCTTCAGGGCTTTCTCGTTC-3′ (reverse); *CTR*, 5′-TCAGGAACCACGGAATCCTC-3′ (forward), 5′-ACATTCAAGCGGATGCGTCT-3′ (reverse); *NFATc1*, 5′-CCGTTGCTTCCAGAAAATAACA-3′ (forward), 5′-TGTGGGATGTGAACTCGGAA-3′ (reverse); *c-fos*, 5′-CCAGTCAAGAGCATCAGCAA-3′ (forward), 5′-AAGTAGTGCAGCCCGGAGTA-3′ (reverse); *β-actin*, 5′-TCCTGTGGCATCCACGAAACT-3′ (forward), 5′-GAAGCATTTGCGGTGGACGAT-3′ (reverse). The relative expression of each target gene was normalized to *β-actin* (*n* = 3).

### 4.12. Western Blot Analysis

BMMs cells pre-treated with or without JH were washed twice with ice-cold PBS and then lysed with radioimmunoprecipitation assay (RIPA) buffer. After centrifugation, the protein in the supernatant was separated by SDS-PAGE electrophoresis, and then transferred to nitrocellulose membranes. After blocking with 5% skim milk for 1 h, the membranes were probed with specific antibodies against phospho-p38, phospho-ERK1/2, phospho-JNK1/2, p38, ERK1/2, JNK1/2, IκB, NFATc1, c-Fos and GAPDH. The antibody reactivity was detected using Enhanced Chemiluminescence (ECL) reagents (Amersham, Shanghai, China) according to manufacturer’s instructions. Three independent experiments were conducted.

### 4.13. Statistical Analysis

The data from each experiment were presented as the mean ± standard deviation (SD) from at least three independent experiments. Statistical comparisons were performed using one-way ANOVA, followed by Tukey’s post hoc analysis. *p* < 0.05 was considered statistically significant.

## 5. Conclusions

In summary, this study elucidated the inhibitory actions of JH on osteoclast formation and function in vitro and attenuated Ti particle-induced osteolysis in vivo. Further mechanism analysis revealed that the inhibition of JH was mediated via the suppression of MAPKs (p38 and ERK) signaling pathways. Our results strongly suggested that JH has a great potential to be developed into a novel therapeutic natural agent for treating periprosthetic osteolysis or other osteoclast-related osteolytic diseases.

## Figures and Tables

**Table 1:** Effects of JH on body weight and serum levels of ALT and AST in Ti particle-induced mice.

Parameters	Control	Vehicle	JH (25 mg∙kg^−1^)	JH (100 mg∙kg^−1^)
Initial weight (g)	22.02 ± 1.30	21.60 ± 0.61	21.99 ± 0.57	21.46 ± 0.41
Final weight (g)	22.29 ± 1.56	22.37 ± 0.42	22.45 ± 0.76	22.94 ± 0.73
AST (U/L)	27.88 ± 1.80	29.37 ± 2.48	22.96 ± 4.47	22.55 ± 3.31
ALT (U/L)	9.99 ± 1.83	8.68 ± 1.84	8.60 ± 0.91	9.06 ± 2.15

Ti particle-induced mice were administrated with different doses of JH (25 mg∙kg^−1^ and 100 mg∙kg^−1^) or PBS daily for up to 14 days. Data are expressed as mean ± SD (*n* = 7). ALT, alanine aminotransferase; AST, aspartate aminotransferase.
